# Outcomes of clinical utility in amyloid-PET studies: state of art and future perspectives

**DOI:** 10.1007/s00259-020-05187-x

**Published:** 2021-02-17

**Authors:** Matteo Cotta Ramusino, Giulia Perini, Daniele Altomare, Paola Barbarino, Wendy Weidner, Gabriella Salvini Porro, Frederik Barkhof, Gil D. Rabinovici, Wiesje M. van der Flier, Giovanni B. Frisoni, Valentina Garibotto, Stefan Teipel, Marina Boccardi

**Affiliations:** 1 Unit of General Neurology, IRCCS Mondino Foundation, Pavia, Italy; 2 Department of Brain and Behaviour, University of Pavia, Pavia, Italy; 3 Laboratory of Neuroimaging of Aging (LANVIE), University of Geneva, Geneva, Switzerland; 4 Memory Clinic, Geneva University Hospitals, Geneva, Switzerland; 5 Alzheimer’s Disease International, London, UK; 6 Alzheimer Italia Federation, Milan, Italy; 7 Department of Radiology and Nuclear Medicine, Amsterdam University Medical Centre, Amsterdam, NL the Netherlands; 8 Queen Square Institute of Neurology and Centre for Medical Image Computing, University College, London, UK; 9 Departments of Neurology, Radiology and Biomedical Imaging, University of California, San Francisco, CA USA; 10 Department of Neurology, Amsterdam Neuroscience, Alzheimer Center Amsterdam, Vrije Universiteit Amsterdam, Amsterdam, The Netherlands; 11 NIMTlab, Neuroimaging and Innovative Molecular Tracers Laboratory, University of Geneva, Geneva, Switzerland; 12 Nuclear Medicine and Molecular Division, Geneva University Hospitals, Geneva, Switzerland; 13 Department of Psychosomatic Medicine, University of Rostock, Rostock, Germany; 14 Clinical Dementia Research Group, German Center for Neurodegenerative Diseases (DZNE), Rostock-Greifswald site, Rostock, Germany; 15 Late Translational Dementia Research Group, German Center for Neurodegenerative Diseases (DZNE), Rostock-Greifswald site, Rostock, Germany

**Keywords:** Outcome, Clinical utility, Amyloid-PET, Diagnostic biomarkers, Alzheimer’s disease, Systematic review

## Abstract

**Purpose:**

To review how outcomes of clinical utility are operationalized in current amyloid-PET validation studies, to prepare for formal assessment of clinical utility of amyloid-PET-based diagnosis.

**Methods:**

Systematic review of amyloid-PET research studies published up to April 2020 that included outcomes of clinical utility. We extracted and analyzed (a) outcome categories, (b) their definition, and (c) their methods of assessment.

**Results:**

Thirty-two studies were eligible. (a) Outcome categories were **clinician-centered** (found in 25/32 studies, 78%), **patient-/caregiver-centered** (in 9/32 studies, 28%), and **health economics-centered** (5/32, 16%). (b) Definition: Outcomes were mainly defined by clinical researchers; only the ABIDE study expressly included stakeholders in group discussions. Clinician-centered outcomes mainly consisted of incremental diagnostic value (25/32, 78%) and change in patient management (17/32, 53%); patient-/caregiver-centered outcomes considered distress after amyloid-pet-based diagnosis disclosure (8/32, 25%), including quantified burden of procedure for patients’ outcomes (*n* = 8) (1/8, 12.5%), impact of disclosure of results (6/8, 75%), and psychological implications of biomarker-based diagnosis (75%); and health economics outcomes focused on costs to achieve a high-confidence etiological diagnosis (5/32, 16%) and impact on quality of life (1/32, 3%). (c) Assessment: all outcome categories were operationalized inconsistently across studies, employing 26 different tools without formal rationale for selection.

**Conclusion:**

Current studies validating amyloid-PET already assessed outcomes for clinical utility, although non-clinician-based outcomes were inconsistent. A wider participation of stakeholders may help produce a more thorough and systematic definition and assessment of outcomes of clinical utility and help collect evidence informing decisions on reimbursement of amyloid-PET.

**Supplementary Information:**

The online version contains supplementary material available at 10.1007/s00259-020-05187-x.

## Introduction

In recent years, numerous steps forward have been made in Alzheimer’s disease (AD) diagnostic procedures. Pathophysiologic diagnostic biomarkers support a diagnosis at early stages of the disease and can detect it even at preclinical stages [1, 2]. However, translating such biomarkers from an experimental setting to daily clinical practice requires evidence of clinical utility [3] and cost effectiveness, to support regulatory approval, clinical recommendations, and reimbursement of the test. Clinical utility can be defined as the impact produced by the use of the diagnostic biomarker on patient-centered health outcomes, such as preventing death, restoring, or maintaining health and well-being. However, giving the complexity, the duration, and the limited array of treatment of AD and related disorders, this target is difficult to operationalize.

**Fig. 1:**
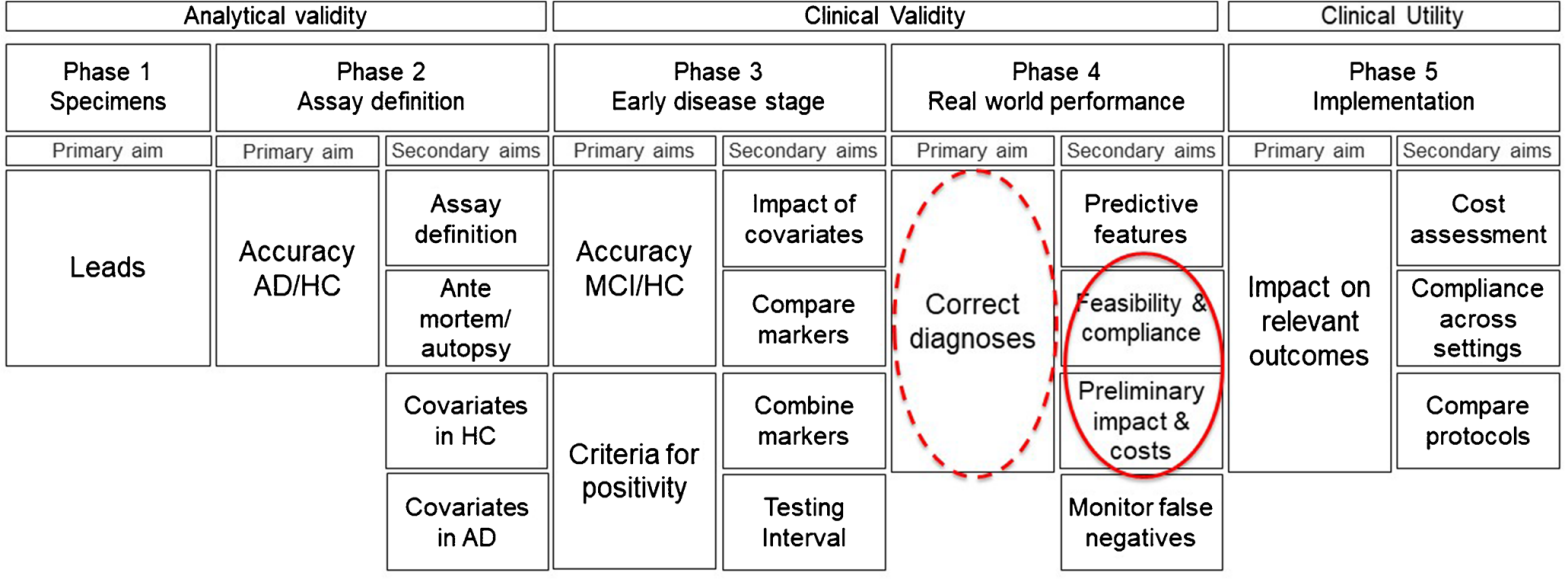
The strategic biomarker roadmap validation phases targeted by the studies included in this review [5, 6]. This figure is a derivative from “The Strategic Biomarker Roadmap for the validation of Alzheimer’s diagnostic biomarkers: methodological update” by M. Boccardi et al [6], used under a Creative Commons Attribution 4.0 International License CC-BY (http://creativecommons.org/licenses/by/4.0/legalcode). The outcomes of interest for this review are those relative to phase 4 and phase 5 that is not yet investigated in amyloid-PET studies. Phase 4 studies require to replicate evidence of clinical validity in real world samples, and allow to collect preliminary information concerning costs and impact on disease burden, thus preparing the methods required for the systematic evaluation of clinical utility in phase 5 studies. Therefore, phase 4 studies include preliminary measures of clinical utility answering secondary aims 2–3 (continuous red circle), and measures of clinical utility sometimes used as surrogate measures of clinical validity to answer the primary aim (red dotted circle): *Phase 4/primary aim target*: To determine the operating characteristics of the biomarker-based diagnostic test in MCI patients in the memory clinics population (replicating the phase 3 accuracy studies in a real-world context). *Phase 4/secondary aim 2*: To assess the practical feasibility of implementing the biomarker-based diagnostic procedure and compliance of test-positive subjects with workup recommendations. (In this aim we also included emotional and social implications related to the positive result disclosure.) *Phase 4/secondary aim 3*: To make preliminary assessments of the effects of biomarker-based diagnosis on costs and burden associated with AD

The 2017 strategic biomarker roadmap provided a methodological framework for the systematic validation of AD diagnostic biomarkers into 5 phases, assessing analytical validity (phases 1–2), clinical validity (phases 3–4), and clinical utility (phase 5) [4, 5] (Fig. 1). While phases 1–3 assess the diagnostic accuracy of biomarkers and define their operating procedures in controlled experimental conditions, phase 4 replicates the studies assessing biomarker clinical validity in real-world contexts. Here, patient cohorts are less strictly selected, and diagnostic protocols may be less rigorously complied with. Importantly, in phase 4 patients are not only diagnosed but also treated according to a diagnosis that is formulated also based on the biomarker under validation. This clinical research context allows collecting preliminary information concerning costs and impact on disease burden and is the proper context to prepare the methods required for the systematic evaluation of clinical utility that will take place in phase 5 studies. The final evidence of clinical utility is eventually required to define clinical guidelines by professional and scientific institutions [2] as well as by other bodies like, for example, Health Technology Agencies, to support policy decision-making concerning the reimbursement of scientific innovations [3, 7, 8].

A definition of clinically meaningful outcomes and of their assessment tools is thus mandatory to achieve conclusive evidence on biomarker utility and should be formulated while performing phase 4 studies, preparing to phase 5 assessments [4]. As a preliminary step to a systematic definition, we provide an overview of how clinical utility is currently operationalized in the ongoing validation studies of amyloid imaging, an AD diagnostic biomarker at advanced development phase.

## Methods

We performed a systematic review of the literature selecting all validation studies of amyloid-PET including outcomes of clinical utility, explored in subjects with mild cognitive impairment or mild dementia. We searched original papers from the Pubmed, Embase, and Cochrane databases and reviews’ references lists. Moreover, we screened two of the largest clinical trial databases (the US National Institute of Health database and the EU Clinical Trials Register from the EMA) to include up-to-date information from studies expected to deliver results shortly.

### Design of the literature review

Since outcomes of clinical utility are preliminarily investigated in phase 4 and more fully in phase 5 of the biomarker roadmap, search strings were set to include and primarily focus on studies belonging to these two phases. The original keywords used to identify articles and clinical trials including amyloid-PET imaging were (“PET” OR “Positron Emission Tomography”) AND (“amyloid” OR “PIB” OR “Pittsburgh compound B” OR “Florbetapir” OR “Flutemetamol” OR “Florbetaben”). In order to include phase 4 studies, the following keywords were added to the original ones: (“clinical diagnosis” OR “memory clinic”) AND (“benefits” OR “outcome” OR “improvement” OR “mortality” OR “morbidity” OR “QoL” OR “quality of life” OR “cost”) AND (“Alzheimer”) AND (“MCI” OR “mild cognitive impairment” OR “prodromal” OR “mild dementia”). Any phase 5 studies could be captured adding the following keywords to the ones of phase 4: (“protocol” OR “recommendation” OR “criteria”) AND (“utility” OR “usefulness” OR “impact” OR “cost” OR “effectiveness”). Keywords were edited according to the strategic biomarker roadmap [5, 6], adapted to amyloid imaging, and the syntax was adjusted for each database (Pubmed, Embase, and Cochrane Library). Reviews’ references lists were also searched for any additional papers. The literature review was replicated independently by two experts in neurodegenerative dementing disorders (MCR and GP), and the non-overlapping papers were merged.

### Eligibility criteria

We assessed papers published up to April 2020 and included those reporting outcomes of clinical utility. Papers including only clinical validity (e.g., diagnostic accuracy compared to a reference standard), but not clinical utility (e.g., practical impact on patients) measures, were excluded. Only articles indexed in English language were included. Reviews, commentaries, opinion pieces, conference papers, and grey literature were excluded. No geographic or sample size limitation was considered. Relative to ongoing projects, we focused on European and International initiatives designed to quantify clinical utility of amyloid-PET in a real-world setting, such as the AMYPAD, IDEAS, and ABIDE studies [9–11].

**Fig. 2:**
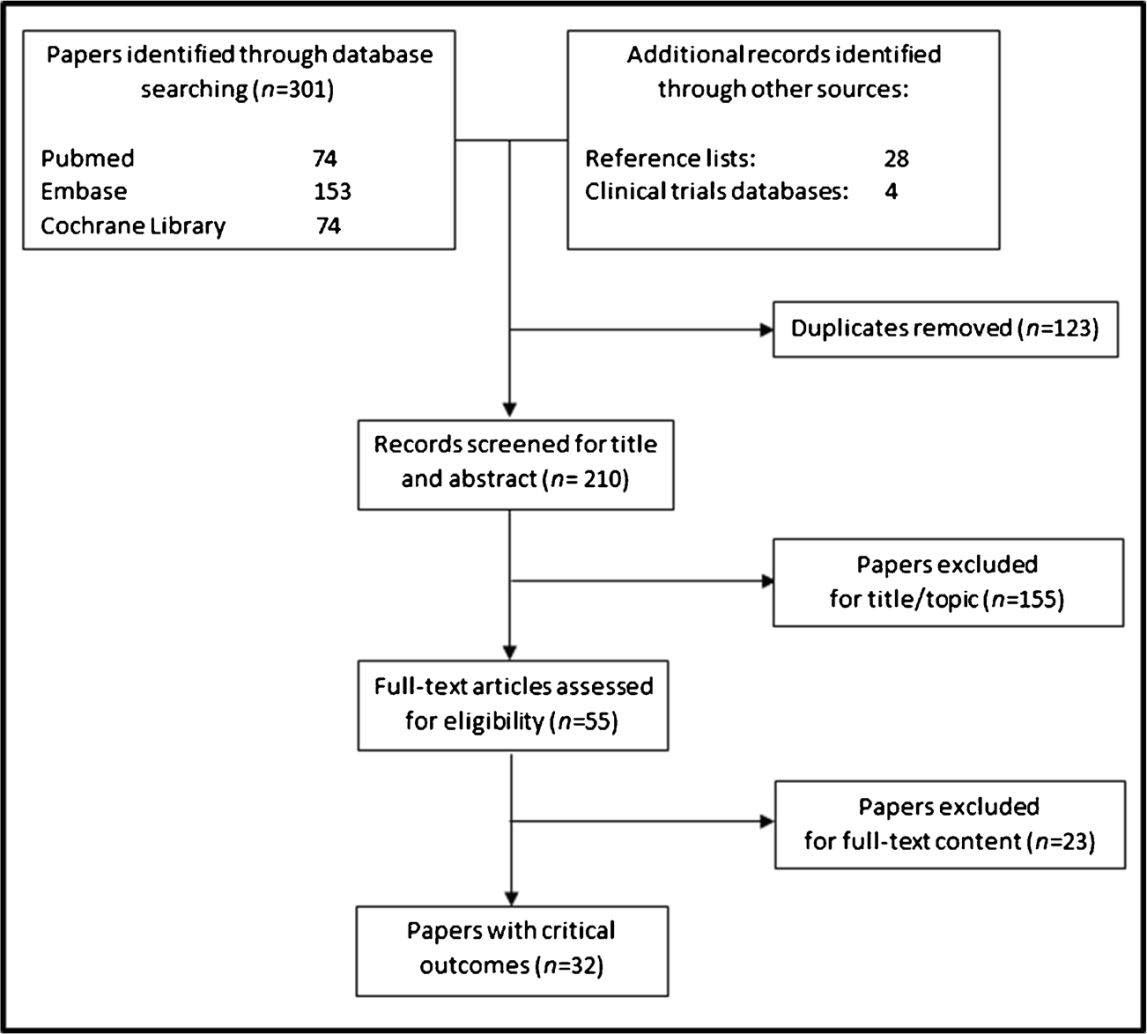
Preferred Reporting Items for Systematic Reviews and Meta-Analyses (PRISMA) flowchart of selected papers

Literature searches have been reported according to the standards of the Preferred Reporting Items for Systematic Reviews and Meta-Analyses (PRISMA) [12] (Fig. 2). From the eligible studies (Table 1), we have extracted the critical variables and processed them to identify (a) specific outcomes of clinical utility, and homogeneous categories thereof (Outcomes domains, Section 3.1); (b) how outcomes were defined, in terms of participation of the pertinent stakeholder to their definition (Outcome definition, Section 3.2); and (c) how outcomes were assessed (Outcome assessment, Section 3.3).

**Table 1:** Phase and aims addressed by the selected studies [5, 6]

First author	Date	Phase	Aim
Frederiksen et al.	2012	4	Primary
Shipke et al.	2012	4	Primary
Guo et al.	2012	4	Secondary 3
Degerman Gunnarsson et al .	2013	4	Primary
Ossenkoppele et al.	2013	4	Primary
Grundman et al.	2013	4	Primary
Mitsis et al.	2014	4	Primary
Sanchez-Juan et al.	2014	4	Primary
Zannas et al.	2014	4	Primary
Hornberger et al.	2015	4	Secondary 3
Ishii et al.	2016	4	Primary
Grundman et al.	2016	4	Primary
Boccardi et al.	2016	4	Primary
Weston et al.	2016	4	Primary
Ceccaldi et al	2016	4	Primary
Bensaїdane et al.	2016	4	Primary, secondary 2
Lim et al.	2016	4	Secondary 2
Carswell et al.	2017	4	Primary
Zwan et al.	2017	4	Primary
Zhong et al.	2017	4	Primary
Pontecorvo et al.	2017	4	Primary
de Wilde et al.	2017	4	Primary, secondary 2
Grill et al.	2017	4	Secondary 2
Vanderschaeghe et al.	2017	4	Secondary 2
Visser et al.	2017	4	Secondary 2
Hornberger et al.	2017	4	Secondary 3
de Wilde et al.	2018	4	Primary
Leuzy et al.	2019	4	Primary
Frisoni et al.	2019	4	Primary, secondary 1, 2, 3
Rabinovici et al.	2019	4	Primary, secondary 1, 2, 3
Triviño-Ibáñez et al.	2019	4	Primary
Cotta Ramusino et al.	2020	4	Primary

## Results

Three hundred and one papers are found from database searching and 32 additional records from cross-references and clinical trials registers (Fig. 2). Comparing the studies identified by the two reviewers, only two papers did not overlap. After title/abstract assessment (155 excluded), 55 articles reported the outcomes of interest and were fully examined. Of these, 23 were excluded for one either not including the population of interest (MCI and/or mild dementia due to AD), not including a clear definition of the outcomes and of the tools used to assess them, for including only outcomes of clinical validity (diagnostic accuracy toward a gold/reference standard), or being reviews or meta-analyses. Finally, 32 studies are eligible (Tables 1 and 2). The 32 studies were published between 2012 and 2020.

**Table 2:** Outcomes of clinical utility for amyloid PET and their measures

Outcomes	Outcome measures	N/tot papers (%)	Reference
Clinician-centered			
-impact on diagnostic procedure	-change in diagnosis (% of cases)	25/32 (78%)	*Frederiksen et al, 2012; Shipke et al, 2012; Degerman Gunnarsson et al, 2013; Grundman et al, 2013; Ossenkoppele et al, 2013; Mitsis et al, 2014; Sanchez-Juan et al, 2014; Zannas et al, 2014; Ishii et al, 2016; Grundman et al, 2016; Boccardi et al, 2016; Bensaїdane et al, 2016; Weston et al, 2016; Ceccaldi et al, 2017; Carswell et al, 2017; Zwan et al, 2017; Zhong et al, 2017; Pontecorvo et al, 2017; de Wilde et al, 2017-2018 (ABIDE project); Leuzy et al, 2019; Frisoni et al, 2019 (AMYPAD study); Rabinovici et al, 2019 (IDEAS study); Cotta Ramusino et al, 2020*
	-gain in diagnostic confidence (on 0-100% scale)		*Frederiksen et al, 2012 ; Shipke et al, 2012; Grundman et al, 2013; Ossenkoppele et al, 2013; Ishii et al, 2016; Bensaїdane et al, 2016; Boccardi et al, 2016; Weston et al, 2016; Ceccaldi et al, 2017; Zhong et al, 2017; Pontecorvo et al, 2017; de Wilde et al, 2017-2018 (ABIDE project); Triviño-Ibáñez et al, 2019; Frisoni et al, 2019 (AMYPAD study); Rabinovici et al, 2019 (IDEAS study); Cotta Ramusino et al, 2020*
	-time to communicate to the patient an etiologic diagnosis with very high confidence (≥90%)		*Frisoni et al, 2019 (AMYPAD study)*
-impact on management	-change in management: • pharmacological change (% of cases) • request for other diagnostic procedures (% of cases) • request for counseling (% of cases) • referral to AD clinical trial (% of cases)	17/32 (53%)	*Shipke et al, 2012; Grundman et al, 2013; Grundman et al, 2016; Boccardi et al, 2016; Bensaїdane et al, 2016; Weston et al, 2016; Pontecorvo et al, 2017; Zwan et al, 2017; Ceccaldi et al, 2017; Zhong et al, 2017; Carswell et al, 2017; de Wilde et al, 2017-2018 (ABIDE project); Triviño-Ibáñez et al, 2019; Leuzy et al, 2019; Frisoni et al, 2019 (AMYPAD study); Rabinovici et al, 2019 (IDEAS study)*
Patient- and caregiver-centered			
-tolerability and impact of amy-PET result disclosure	-change in the perceived risk of developing dementia due to AD (on 0-100% scale or Mishel Uncertainty in Illness scale-MUIS)^a^ -Memory Complaints Questionnaire (MAC-Q)^a^ -Impact of event scale-revised (IES-R)^a^ -Geriatric Depression Scale (GDS)^a^ -Depression, Anxiety, and Stress Scale (DASS)^a^ -Beck Anxiety Index (BAI)^a^ -State-Trait Anxiety Inventory (STAI)^a^ -Euro Qol - 5 Dimension (EQ-5D)^a^ -Quality of Life – Alzheimer’s Disease (QoL-AD)^a^ -ICEpop CAPability (ICECAP) assessment^a^	6/32 (19%)	*Lim et al, 2016; Pontecorvo et al, 2017; Grill et al, 2017; Vanderschaeghe et al, 2017; de Wilde et al, 2017 (ABIDE project); Frisoni et al, 2019 (AMYPAD study)*
-burden of the procedure	-overall judgement (yes/no) and list of main causes (i.e adverse effects)	1/32 (3%)	*de Wilde et al, 2017 (ABIDE project)*
-active participation in decision-making process	-Observing PaTient InvOlvemeNt scale (OPTION12)^a^ -Control Preferences Scale (CPS)^a^ -Shared Decision Making Questionnaire (SDM-Q-9)^a^	1/32 (3%)	*Visser et al, 2019 (ABIDE project)*
- impact on caregivers	-caregiver questionnaires (based on Likert scale) -Resource Utilization in Dementia Questionnaire Scale (RUD)^a^ -Zarit Burden interview (ZBI)^a^ -Self Efficacy for Managing Dementia^a^ -Brief Cope Assessment^a^	3/32 (9%)	*Bensaїdane et al, 2016; Pontecorvo et al, 2017; Frisoni et al, 2019 (AMYPAD study)*
- survival	-mortality rate	1/32 (3%)	*Rabinovici et al, 2019 (IDEAS study)*
Health economics-centered			
-effects on prognosis	-quality-adjusted life years (QALYs) -time to institutional care	1/32 (3%)	*Guo et al, 2012*
-costs	-incremental cost-effectiveness ratios (ICERs) -use of healthcare resources (e.g. diagnostic tests, hospital admissions and visits), and number of patients who can be discharged from the memory clinic (saving in terms of assistance and care) -costs of diagnostic workup to the etiologic diagnosis with very high confidence (≥90%) -caregiver time -mortality	5/32 (16%)	*Guo et al, 2012; Hornberger et al, 2015-2017; Frisoni et al, 2019 (AMYPAD study); Rabinovici et al, 2019 (IDEAS study)*

^a^Short descriptions of the scales are reported in the supplementary table.

### Outcome domains

Based on the issue examined in the papers, we have identified three main outcome domains: (a) *clinician-centered outcomes*, quantifying the impact on the diagnostic procedure in terms of physician’s diagnostic confidence and patient management; (b) *patient- and caregiver-centered outcomes*, operationalizing the ultimate target of patients benefit; and (c) *health economics-centered outcomes*, consisting either of costs alone or of combined evidence of costs and other impacts on health resource utilization (Table 2). (a) Most of the selected studies (25/32, 78%) [9, 11, 13–35] included outcomes belonging to the clinician-centered domain and assessed the incremental diagnostic value for physicians, e.g., in terms of diagnostic change and increased diagnostic confidence (25/32, 78%), and changes in the planning of patient management (17/32, 53%) (Table 2). Amyloid-PET led to diagnostic revision in 19–79% of cases, to increased diagnostic confidence (9–49%), and to revise management plans (24–89% of cases) [13–35]. (b) Patient- and caregiver-centered outcomes are reported in 28% (9/32) [9, 11, 24, 30, 33, 36–39] of the studies and mainly assessed the patients’ and caregivers’ distress after amyloid-pet-based diagnosis disclosure (8/32, 25%) (Table 2). In particular, patient-centered outcomes were more heterogeneous than clinical-centered ones: most (6/8, 75%) quantified the psychological burden from the result disclosure [9, 11, 30, 36–38], 1/8 (12.5%) assessed the burden of the mere technical procedure [11], and 1/8 (12.5%) assessed the patient’s participation in decision-making [39] (Table 2). Patient-centered outcomes mainly provided evidence immediately related to biomarker use (e.g., the psychological implications of the procedure for the patient 75%) [9, 11, 30, 36–38]. Only one study provided measures of mortality rate in patients diagnosed with or meant to undergo amyloid-PET [33]. Summarizing, patients reported relevant advantages after the biomarker disclosure, such as a greater awareness about their health status and the possibility of making practical and medical arrangements, but also some disadvantages, such as the development of mood disorders, feeling worried about when their symptoms might worsen, and about the risk of a patronizing attitude by relatives [30, 33, 36–39]. (c) Finally, health economics-centered outcomes are included in 16% (5/32) [9, 33, 40–42] of the studies and assessed the costs in terms of time and healthcare resources spent to achieve a high-confidence etiological diagnosis (5/32, 16%) and preliminarily impact on quality of life and prognosis (1/32, 3%) (Table 2). Amyloid-PET was estimated to increase mean life expectancy, quality-adjusted, by 0.008–0.150 years compared to patients undergoing the usual diagnostic workup, with cost savings of around $ 12,500 per patient over lifetime in medical care [40–42].

### Outcome definition

Overall, outcomes were defined by clinical researchers, resulting occasionally from a discussion with regulators (EMA and FDA were involved in the design of the Amypad [9] and IDEAS [10] studies, respectively), and in one case from an active participation of patients and caregivers, namely in the ABIDE study [11]. This European project analyzed audiotaped discussion groups and consultations where patients and caregivers were involved in deciding which diagnostic test to perform and then discussing test results [11].

Mortality was considered only in the IDEAS study, where it was defined as pre- and post-procedural death rate [33].

### Outcome assessment

The patient-, caregiver-, and health economics-centered outcomes are inconsistently operationalized across studies, and the tools used to measure them were heterogeneous (26 different tools, Table 2). No formal criteria or consensus procedures to select or prioritize the assessment tools were reported in the examined papers.

## Discussion

In this paper, we reviewed which outcomes of clinical utility were assessed in current validation studies of amyloid-PET [4, 5, 43], one of the most advanced diagnostic biomarkers for AD, and how they were defined and measured. The outcomes of clinical utility assessed in the eligible studies were mostly confined to diagnostic utility for clinicians, patient tolerability of disclosure of results, and costs of the amyloid-PET-based diagnosis. These outcomes provide a first operationalization of clinical utility for amyloid-PET; such operationalization is required to complete phase 4 studies of clinical validity, and to prepare to phase 5 studies, where the actual cost-weighted health benefit will be assessed (Fig. 1). Our review also allows to identify aspects and dimensions that should be improved in order to design sound phase 5 studies, aimed to quantify the impact of the use of diagnostic biomarkers on society in terms of improved health and cost-effectiveness, thus generating the full array of information required for future evidence-based clinical and policy decision making. Previous reviews in the field focused on the measures of utility within the diagnostic procedure only [44–51]. Relative to this previous work, we adopted a wider perspective, aiming to identify the definition and operationalization of clinical utility in view of designing phase 5 studies. This methodological overview is therefore necessary to support the next validation steps for AD diagnostic biomarkers.

### Domains and specific outcomes

#### Clinician-centered outcomes

Amyloid-PET validation is now in phase 4, entailing studies based on real-world patients, where the biomarker is not only assessed, but also used to support diagnosis and treatment [4, 5]. Reflective of this, preliminary outcomes of impact are becoming to be available (Table 2), though mostly confined to outcomes assessing the utility of the exam during the diagnostic workup (clinician-centered outcomes, 78%) [9, 11, 13–35]. This outcome domain is indeed the first that can be collected as clinical innovations enter phase 4 validation studies. On the other hand, the connection of an improved diagnosis to the final impact on patient health and other ultimate targets of clinical utility remains to be demonstrated and operationalized. Indeed, the lack of a gold standard (e.g., pathology,or clinical diagnosis at follow-up) does not allow to ascertain whether these outcomes correspond really to a more correct diagnosis or to a more appropriate treatment and therefore to a better medical care. Moreover, the early appropriate diagnosis is only the first step toward a possible improvement of the patient’s health and quality of life, which should be compared through RTCs comparing concrete outcomes of health, quality of life, and costs in patients who did and did not undergo amyloid-PET-based diagnosis.

*Patient- and caregiver-centered outcomes*, reported in approximately one-third of the eligible studies [9, 11, 24, 30, 33, 36–39], were heterogeneous: they included the burden of the technical procedure [11], patient participation in decision-making [39], and the impact of result disclosure [9, 11, 30, 36–38]. Understandably, and similar to the clinician-centered outcomes, these are directly related to the diagnostic procedure and therefore the first to be assessed in phase 4 studies. By design, the ultimate target outcomes of patient health and well-being are left unexplored in such studies. However, although these will be systematically assessed later on, phase 4 is already meant to start defining them. The dimension that are not yet present in those studies includes quality of life, disability, mortality, and caregiver-relevant outcomes, such as those related to the management of the behavioral and psychological symptoms of dementia. Operationalizing these outcomes is not a trivial task. Previous projects aiming to this target, i.e., the “Dementia Outcomes Measurement Suite” (DOMS) [52] and the “Real world Outcomes across the Alzheimer’s Disease spectrum for better care: Multi-modal data Access Platform” (ROADMAP) [53], also found gaps in these key areas of Alzheimer’s disease and related disorders. In particular, they showed a lack in the operationalization of outcomes related to the final stage of the disease, entailing relevant long-term outcomes [54, 55]. Nonetheless, the experience gathered in such previous projects should be leveraged to move the next steps forward.

*Health economics-centered* outcomes were reported in only 16% of the eligible studies [9, 33, 40–42] but are being assessed more exhaustively in ongoing clinical trials [9, 10]. The health economics-centered outcomes extracted by our review addressed costs and benefits immediately related to the diagnostic procedure, e.g., the costs of the diagnostic workup required to achieve a high-confidence etiological diagnosis [9, 33, 40–42] and the cost-saving on further diagnostic investigations and specialist visits [9, 33]. Some studies also tried to quantify the impact of amyloid-PET on patient prognosis in terms of quality-adjusted life years or institutionalization rate, but this was done through statistical models (state transition probability analyses and simulation models [40–42]) rather than on collected data. Future longitudinal studies collecting evidence from all those impacted by the disease (e.g., relatives, caregivers) will provide firmer evidence; however, these models can inform the choices on the definition, selection, and assessment of target outcomes. So far, anyway, a comprehensive definition of patient- and caregiver-centered outcomes, including the health- and burden-related measures for both, is still lacking formally and should be defined in the short term also to properly operationalize and quantify the different disease-related costs, including those less easily monetizable [42].

### Definition of the identified outcomes

Overall, outcomes have been mainly defined by clinical researchers, without a strategy of explicit and active participation among stakeholders. The only exception to this procedure was the ABIDE study [11]. In ABIDE, clinicians, patients and caregivers gathered in focus groups, expressed their views, experiences and perceived dilemmas regarding diagnostic testing and communication of test results. Their audiotaped discussions were then used to extract recommendations to effectively involve patients and caregivers in deciding about diagnostic testing and to best discuss test results. In this way, researchers managed for the first time to engage these parties in the decision-making process, exploring new communication ways and sharing strategies. The explicit aim was to expand the point of view of clinical researchers, emerging as dominant also from the results of this review. The ABIDE project may thus inspire a more balanced involvement of the main stakeholders, for a definition of outcomes better complying with societal needs as well as regulators requirements.

The IDEAS project assessed payer-relevant outcomes like hospitalization and mortality in a group of patients meant to undergo amyloid-PET [33]. Mortality was expressed as number of deaths occurred within this group 1 month before and 3 months after the planned/performed PET scan, in a non-randomized longitudinal study design. Many AD-relevant outcomes are temporally distant from biomarker acquisition (e.g., disability, institutionalization); mortality is certainly the most distant, but no less relevant. An early biomarker-based diagnosis could indeed allow for a quicker access to appropriate pharmacological and non-pharmacological treatments, as well as a better healthcare, and thus positively affect the progression of disease and the long-term outcomes, such as institutionalization and possibly death. To make such an assessment feasible, next steps may consist of identifying intermediate outcomes demonstrated to be significantly connected to mortality and use them as proxies to compute the impact of the diagnostic procedure. Moreover, similar to health economics outcomes, years of life may not be meaningful per se but should be complemented by quality of life measures (QALYs).

### Outcome assessment

Patient- and caregiver-centered outcomes were operationalized inconsistently across studies and consequently assessed with heterogeneous tools. On the other hand, these tools consist of widely available and easy-to-administer questionnaires and scales (Table 2). Their main limitation lies in their lack of validation across cultures. Cultural adaptation is required to guarantee proper assessment, reproducibility, and comparability of results [56]. Patient and caregiver participation to outcome definition and prioritization would also help identify the best tools, ensuring effective quantification of patient-/caregiver-relevant outcomes, including psychological and physical well-being. Other relevant issues are also being taken into account, like gender-related features, or outcomes related to a quickly evolving digitally supported world [57].

### Next steps and future perspectives

AD diagnostic biomarkers support an accurate and early etiological diagnosis, which is considered a priority not only by clinicians but also by global policies (global action plan on the public health response to dementia 2017–2025 [58], and World Alzheimer Report 2011 [59]). The possible availability of disease-modifying treatments should not be the only reason in support of an early diagnosis. Indeed, an early detection allows access to treatments of proven efficacy, both pharmacological (e.g., acetylcholinesterase inhibitors) and non-pharmacological (e.g., cognitive rehabilitation) [60, 61]; guarantees more proper handling of behavioral and psychiatric symptoms in those with more advanced disease stage [62, 63]; allows access to clinical trials; and facilitates decisions on life arrangements [64]. However, to date, the utility of an early detection still cannot emerge from available data [65], which may prevent the implementation of these diagnostic procedures as well as the pursuing of the above global priorities. On the other hand, promoting clinical biomarker testing before knowing its actual cost-effectiveness may expose to possible overdiagnosis (e.g., in the case of asymptomatic individuals positive to brain amyloidosis). If useful for research purposes, the potential harm provided to patients [66] should be weighted thanks to thorough assessment of pertinent outcomes. Improved outcome definition may therefore allow more sensitive detection of both potential positive and negative effects of early diagnosis, while better informing about any significant increase of patient health as required by regulators and policy makers to decide on the approval and reimbursement of new diagnostic procedures [3, 7, 8]. Researchers in other fields of medicine are facing this issue, as well. For example, disciplines like oncology, psychiatry, or other diagnostic fields, such as imaging, laboratory, or genetic testing, also suffer from immature outcome definition, attributed to a lack of a consented definition of clinical meaningfulness [67, 68]. The assessment of clinical utility in these fields is, as for amyloid-PET, limited to the impact of the biomarker-based diagnostic procedure on prescribed treatment and on patients’ psychological reaction to diagnosis disclosure, besides measures of survival more frequently assessed in oncology. Conversely, measures assessing patient social functioning and autonomy in daily life, as well as measures of caregiver overall well-being, are similarly scanty. This limitation depends crucially on the lack of a consensual and comprehensive definition of clinical meaningfulness in the first place, and on its challenging operationalization. Indeed, the concept of clinical meaningfulness is complex and heterogeneous in itself, including disease-, physical-, and psychosocial-related outcomes [69]. For neurocognitive disorders, this means including not only cognitive and functional outcomes but also emotional, social, and behavioral implications for patients, caregivers, and society [70, 71]. Defining and operationalizing such outcomes require full awareness of the impact that a diagnosis of dementia generates in the family, in the community, as well as in clinical contexts (World Alzheimer Report 2019 [72]). Issues like personal interaction with patients, impact of diagnosis communication, counseling and support along the diagnostic procedure, and continuity in the taking charge of the patient should all be entailed by a consistent and participatory approach. This means that the perspective of treatment should take into account not only the presence or absence of a disease modifier for AD within a uniquely medical perspective but should also include other therapeutic options [60–63], a plan for patient support from and beyond diagnosis (e.g., from plan to impact III [73]), and specific tools facilitating communication between patients, caregivers, and professionals (e.g., CO-desiGning DemeNtia dIagnoSis ANd post-diagnostic CarE, COGNISANCE study [74]). Moreover, outcome assessment should be specific to the social, cultural, and economic context to which it is addressed. This allows to quantify and maximize the health benefits independently on the availability of resources (e.g., strengthening responses to dementia in developing countries, STRiDE [75]) but also to leverage on each other experience and export models and tools to transfer clinical innovation into practice. Such wider framework can provide the context to empower the community in participating more actively in clinical decisions as well as in specific research steps that require their input, in view of serving their own ultimate well-being.

### Limitations

The main limitation of this review consists of considering only the field of amyloid imaging. Other biomarkers are available, at variable status of validation advancement. We chose amyloid-PET for its relatively advanced and well-structured validation. Besides other biomarkers, also other fields, like non-pharmacologic intervention, or other medical fields may provide useful input feeding the next steps toward defining clinical utility, outcomes, proxies, and causal links. Finally, the study population considered in our review consists of MCI and mild dementia. This choice is limited in that results relate to outcomes relevant to or assessable at the early stages of disease. However, this is in line with the purpose of etiological biomarkers to provide a timely diagnosis possibly enabling to delay the development of dementia. Moreover, considering MCI and early dementia allows to assess progression, another possible outcome that can be measured and compared between diagnostic procedures.

## Conclusions

This review describes the current scenario and may help outline future directions to improve the definition of outcomes of clinical utility. More concretely, the availability of different biomarkers (PET-, CSF-, or blood-based) could allow for a better detection rate and diagnostic accuracy, in agreement with the WHO Global Action Plan (Aim 4, [58]), and translate into a more appropriate patient management. The actual impact on health will have to be verified in terms of improved health, quality of life, survival, and costs. In this perspective, next steps require that patient-centered outcomes, focusing on long-term well-being, be better defined and introduced for pilot assessment in phase 4 studies, preparing to proper phase 5 assessments [4, 5]. Long-term outcomes like mortality and disability are relevant and objective, but their link to the diagnostic procedure is difficult to quantify. A way forward in these cases may include (a) the definition of proxies, i.e.. variables that are easier to assess and related to the target outcome and closer in time to the diagnostic procedure, and (b) the demonstration of causal connection between proxies, indicators or other outcomes that lie between the diagnostic procedure and the ultimate target outcome in logical and temporal sequence [76]. Then, models as those used already, but fed with concrete data on outcomes specifically defined a priori, may produce better informed predictions than currently possible [40–42]. The definition and prioritization of such proxies should include a balanced involvement of different pertinent stakeholders in order to effectively respond to the societal needs and provide balanced information for clinical and policy decisions [7, 8]. This collaborative framework should include different agencies (e.g., including health technology ones), so that fine-tuning along the process could spare unwarranted efforts.

## Supplementary Information

ESM 1 (DOCX 18 kb).

## Data Availability

All data generated or analyzed during this study are included in the present paper or in otherwise accessible sources (published papers, project descriptions) referenced throughout.
