# Supplementary material for: Outcomes of clinical utility in amyloid-PET studies: state of art and future perspectives
Source: Eur J Nucl Med Mol Imaging. 2021 Feb 17;48(7):2157–68. doi: 10.1007/s00259-020-05187-x (PMC8175294; doi:10.1007/s00259-020-05187-x)
Supplement: Supplementary file 1 — (DOCX 18 kb). [file 259_2020_5187_MOESM1_ESM.docx]

**Supplementary Table.** Scales and questionnaires used to operationalize patient- and caregiver-centered outcomes.

| Acronym | Name | Short description | Reference |
| --- | --- | --- | --- |
| MUIS | Mishel Uncertainty in Illness Scale | MUIS is a 30-item scale measuring four dimensions of uncertainty (ambiguity, complexity, inconsistency and unpredictability) in symptomatology, diagnosis, treatment, relationship with care-givers | *Mishel, 1981* |
| MAC-Q | Memory Complaints Questionnaire | MAC-Q is a self-reporting questionnaire for elderly people including 6 items: 5 questions addressing changes in daily living, and 1addressing subjective memory complaints, comparing the administration moment to when the person was 20 years old. | *Crook et al, 1992* |
| IES-R | Impact of event scale-revised | IES’s a 22-item self-report measure that assesses subjective distress caused by traumatic life events. The IES-R contains seven additional items related to the hyperarousal symptoms of Post-traumatic Stress Disorder. | *Weiss and Marmar, 1997* |
| GDS | Geriatric Depression Scale | GDS is a 30-item self-report assessment used to identify depression in the elderly | *Yesavage et al, 1982* |
| DASS | Depression, Anxiety, and Stress Scale | DASS is a 42-item self-report instrument designed to measure three negative emotional states: depression, anxiety and tension/stress. | *Lobivond and Lobivond, 1995* |
| BAI | Beck Anxiety Index | BAI is a 21-question multiple-choice self-report inventory that is used for measuring the severity of anxiety. | *Beck et al, 1988* |
| STAI | State-Trait Anxiety Inventory | STAI is a psychological inventory based on a 4-point Likert scale and has 40 items for assessing two types of anxiety: 20 items for the state anxiety, or anxiety about an event, and 20 items for trait anxiety, or anxiety level as a personal characteristic. | *Spielberger et al, 1987* |
| EQ-5D | Euro Qol - 5 Dimensions | In EQ-5D, health status is measured in terms of five dimensions (5D): mobility, self-care, usual activities, pain/discomfort, and anxiety/depression. Each dimension includes three severity levels. | *Brooks et al, 2003* |
| QoL-AD | Quality of Life – Alzheimer’s Disease | QOL-AD is a brief, 13-item measure designed to rate the patient’s quality of life from the patient himself and the caregiver. It considers a variety of life domains, including the patient's physical health, mood, relationships, activities, and ability to complete tasks | *Logsdon et al, 1999* |
| ICECAP | ICEpop CAPability | ICECAP assesses the wellbeing defined in a broader sense, rather than health, including: attachment (love and friendship); security (thinking about the future without concern); role (doing things that make you feel valued); enjoyment (enjoyment and pleasure); and control (independence). | *Coast et al, 2008* |
| OPTION12 | Observing PaTient InvOlvemeNt scale | OPTION is a 12-item scale that measures the level of patient involvement in doctor-patient consultations about treatment choices | *Elwyn et al, 2005* |
| CPS | Control Preferences Scale | CPS is a widely used instrument designed to measure a patient's preferred level of their own versus their doctor's control over a treatment decision | *Degner et al, 1997* |
| SDM-Q-9 | Shared Decision Making Questionnaire | SDM-Q-9 is 9-item self-reported questionnaire designed to  assess the extent to which clinicians involve patients in decision-making | *Kriston et al, 2010* |
| RUD | Resource Utilization in Dementia | RUD is the most widely used instrument to measure the time actually spent by the caregivers in the nursing home on personal and instrumental activities of daily living, and supervision. | *Wimo and Nordberg, 2007* |
| ZBI | Zarit Burden interview | ZBI is an assessment tool for evaluating care-related burden  including caregiver’s health, personal  and social life, financial situation, emotional wellbeing  and interpersonal relationships. | *Zarit et al, 1980* |
| - | Self-Efficacy for Managing Dementia | This scale includes five items originally designed to determine how family caregivers can manage their relatives’ dementia symptoms. | *Fortinsky et al, 2002* |
| - | Brief Cope Assessment | This 28-item self-report questionnaire was designed to measure effective and ineffective ways to cope with a stressful life event. | *Cooper et al, 2008* |
| **Reference:** *(alphabetical order)*  Beck AT, Epstein N, Brown G, Steer RA. An inventory for measuring clinical anxiety: psychometric properties. J Consult Clin Psychol. 1988;56:893–7.  Brooks R, Rabin R, de Charro F. The Measurement and Valuation of Health Status using EQ-5D: A European Perspective. Dorderecht, Kluwer. 2003.  Coast J, Peters TJ, Natarajan L, Sproston K, Flynn T. An assessment of the construct validity of the descriptive system for the ICECAP capability measure for older people. Qual Life Res 2008; 17: 967–76  Cooper C, Katona C, Livingston G. Validity and reliability of the brief COPE in carers of people with dementia: The LASER-AD Study. J Nerv Ment Dis. 2008 Nov;196(11):838-43. doi: 10.1097/NMD.0b013e31818b504c.  Crook TH, Feher EP, Larrabee GJ. Assessment of Memory Complaint in Age-Associated Memory Impairment: The MAC-Q Int Psychogeriatr. Fall 1992;4(2):165-76. doi: 10.1017/s1041610292000991.  Degner LF, Sloan JA, Venkatesh P. The control preferences scale. Can J Nurs Res. 1997;29:21–43.  Elwyn G, Hutchings H, Edwards A, Rapport F, Wensing M, Cheung WY. The OPTION scale: measuring the extent that clinicians involve patients in decision-making tasks. Health Expect. 2005;8:34–42.  Fortinsky RH, Kercher K, Burant CJ. Measurement and correlates of family caregiver self-efficacy for managing dementia. Aging Ment Health. 2002 May;6(2):153-60. doi: 10.1080/13607860220126763.  Kriston L, Scholl I, Holzel L, Simon D, Loh A, Harter M. The 9-item Shared Decision Making Questionnaire (SDM-Q-9). Development and psychometric properties in a primary care sample. Patient Educ Couns. 2010;80:94–99.  Lovibond PF, Lovibond SH. (1995). The structure of negative emotional states: Comparison of the Depression Anxiety Stress Scales (DASS) with the Beck Depression and Anxiety Inventories. Behaviour Research and Therapy, 33, 335-343  Logsdon RG, Gibbons LE, McCurry SM, Teri L. Quality of life in Alzheimer's disease: patient and caregiver reports. J Ment Health Aging. 1999;5:21–32.  Mishel MH. The measurement of uncertainty in illness. Nurs Res. 1981;30(5):258-263.  Spielberger CD, Gorsuch RL, Lushene R, Vagg PR, Jacobs GA. Manual for the State-Trait Anxiety Inventory. Palo Alto, CA: Consulting Psychologists Press; 1983.  Weiss DS, Marmar CR. The impact of event scale – revised. In: Wilson JP, Keane TM, editors. Assessing psychological trauma and PTSD. New York: Guilford Press; 1997. pp. 399–411.  Wimo A, Nordberg G. Validity and reliability of assessments of time: comparisons of direct observations and estimates of time by the use of the Resource Utilization in Dementia (RUD) instrument. Arch Gerontol Geriatr. 44: 2007; 71–81  Yesavage A, Brink TL, Rose TL, Lum O, Huang V, Adey M, Leirer VO. Development and Validation of a Geriatric Depression Screening Scale: A Preliminary Report J Psychiatr Res. 1982-1983;17(1):37-49. doi: 10.1016/0022-3956(82)90033-4.  Zarit SH, Reever KE, Bach-Peterson J. Relatives of theimpaired elderly, correlates of feelings of burden. Gerontologist 1980; 20: 649–6 | | | |
